# Retinal Docosahexaenoic Acid Is Significantly Reduced in Diabetic Humans and Mice: Possible Relationship to Diabetic Retinopathy

**DOI:** 10.1167/iovs.65.14.39

**Published:** 2024-12-27

**Authors:** Dhavamani Sugasini, Poorna C. R. Yalagala, Jason C. Park, Guangying Ma, Zeenat Farooq, Basma Baccouche, Onkar B. Sawant, J. Jason McAnany, Xincheng Yao, Andrius Kazlauskas, Brian T. Layden, Papasani V. Subbaiah

**Affiliations:** 1 Division of Endocrinology, Diabetes and Metabolism, Department of Medicine, University of Illinois at Chicago, Chicago, Illinois, United States; 2 Department of Ophthalmology and Visual Sciences, University of Illinois at Chicago, Chicago, Illinois, United States; 3 Department of Biomedical Engineering, University of Illinois at Chicago, Chicago, Illinois, United States; 4 Center for Vision and Eye Banking Research, Eversight, Cleveland, Ohio, United States; 5 Department of Physiology and Biophysics, University of Illinois at Chicago, Chicago, Illinois, United States; 6 Jesse Brown VA Medical Center, Chicago, Illinois, United States

**Keywords:** omega 3 fatty acids, diabetic retinopathy, docosahexaenoic acid (DHA), retinal thickness, electroretinogram (ERG) b-wave

## Abstract

**Purpose:**

The retina contains the highest concentration of the omega 3 fatty acid, docosahexaenoic acid (DHA), in the body. Although epidemiologic studies showed an inverse correlation between the consumption of omega 3 fatty acids and the prevalence of diabetic retinopathy, there are no data showing the effect of diabetes on retinal DHA in humans. In this study, we measured the DHA content of the retina in diabetic and non-diabetic humans as well as mice and determined the effect of diabetes on retinal thickness and function in mice.

**Methods:**

Fatty acid composition was determined by gas chromatography/mass spectroscopy. Retinal thickness in mice was measured by optical coherence tomography and retinal function was measured by electroretinogram (ERG). Expression of selected genes involved in inflammation and lipid metabolism was determined by quantitative real-time PCR (qRT-PCR).

**Results:**

We found a 40% reduction of DHA in peripheral retina and a 25% reduction in the macula of diabetic humans compared with nondiabetic controls. There was a 24% reduction in retinal DHA of type 2 diabetic mice (db/db) compared with the controls (db/+). The retinal thickness was significantly decreased in db/db mice, especially in the inner retina, and the ERG b-wave amplitudes were significantly attenuated. Increased expression of proinflammatory genes was observed in both human and mouse diabetic retinas.

**Conclusions:**

Retinal DHA is reduced in diabetic humans and mice, which is associated with a thinning of retina and functional defects in diabetic mice. Enriching retinal DHA through diet may be beneficial in the prevention and treatment of diabetic retinopathy.

Docosahexaenoic acid (DHA) is the most abundant fatty acid in the retina of humans and other mammals, constituting up to 60% of the membrane fatty acids in the rod outer segment membranes. It is a natural anti-inflammatory, anti-angiogenic, and anti-vasoproliferative molecule that is essential for the normal functioning of the retina.^1^^–^^3^ Epidemiologic studies show that higher consumption of omega 3 fatty acids is associated with decreased risk of several retinal diseases, including age-related macular degeneration,^4^ glaucoma,^5^ and diabetic retinopathy.^6^^,^^7^ In nonhuman primates, DHA deficiency in prenatal and postnatal periods results in reduced visual acuity, abnormal retinal function, and low retinal and brain DHA levels.^8^^,^^9^ Conversely, infants fed formula containing DHA show significantly higher visual acuity compared with those receiving no DHA supplement.^10^ Furthermore, supplementation with high doses of DHA and xanthophyll has been reported to significantly increase macular sensitivity and integrity in patients with diabetes with nonproliferative retinopathy.^11^ Whereas all these studies suggest that retinal DHA is protective against the development of retinopathy, the effect of diabetes on retinal DHA has not been investigated in humans, although reductions in the erythrocyte DHA and arachidonic acid (ARA) levels have been reported.^12^ Tikhonenko et al.^13^ reported that there is a 28% reduction in retinal DHA in a mouse model of type 2 diabetes.

In this study, we examined the fatty acid composition of the human peripheral retina and macula to investigate whether the high prevalence of retinopathy in patients with diabetes is related to a possible deficiency of retinal DHA. In addition, we analyzed the fatty acid composition of the retina in a mouse model of type 2 diabetes (db/db mouse) and studied the effect of diabetes on retinal thickness and function. These results show that diabetes leads to a significant reduction in the retinal DHA content of retina in humans and mice. Furthermore, the reduced content of retinal DHA was associated with a significant impairment in the electroretinogram (ERG) b-wave and a significant reduction in retinal thickness in diabetic mice.

## Materials and Methods

Peripheral retina and macula samples from seven diabetic and eight non-diabetic donors (anonymized) were obtained from the Eversight Eye Bank. The clinical characteristics of the donors are shown in the Table. All research using ocular tissues from research-consented cadaveric donors was performed in compliance with the guidelines of ARVO Best Practices for Human Eye Research. For the separation of the macula, the posterior eye cups were flattened by creating four cuts through all layers of the eye. A brown-yellow macular region was identified, and an appropriate 3 to 4 mm macular sample was collected. Peripheral retinal samples were collected from the non-central fundus region.

**Table.** Characteristics of Retina and Macula Donors

	Diabetic	Non-Diabetic
Number	7	8
Gender	4 men and 3 women	5 men and3 women
Race	All White	All White
Age, y	61.6 ± 5.7	61.9 ± 9.9
Duration of diabetes	3–40 y	–
Death to preserve time	2 h–12 h	8 h–17 h

Male db/db mice (BKS,Cg,Dock7 Lepr<db>/J-00642) and their non-diabetic (db+) littermates were obtained at the age of 4 weeks and were maintained on normal laboratory chow and acidified water. ERG measurements were performed at 16 weeks and 32 weeks of age, and optical coherence tomography (OCT) measurements were performed at 32 weeks of age. Body weights were measured weekly, and blood glucose measurements were performed once a month. The body weights (g) were 55.8 ± 8.6 g for db/db mice and 28.6 ± 2.4 gm for db/+ mice. Blood glucose values were 446.4 ± 121.3 mg/dL for db/db mice and 137.6 ± 19.0 mg/dL for db/+ mice. The animals were euthanized at 32 weeks of age for analysis of the brain and retina for the fatty acid composition and gene expression studies. All animal protocols were approved by the UIC Animal Care Committee, and all the procedures were carried out in accordance with the ARRIVE guidelines.

### Optical Coherence Tomography 

A custom made OCT equipment was used to measure retinal thickness in mice, as described previously.^14^^,^^15^ One three-dimensional (3D) volume data was recorded for each retinal region, such as the optic nerve head (ONH), dorsal, temporal, ventral, and nasal quadrants. Each volume had a field of view with a diameter of approximately 800 µm. The retinal thickness was measured after manual segmentation. The inner retina thickness was measured from the nerve fiber layer (NFL) to the outer plexiform layer (OPL), and the outer retina thickness was measured from the ONL to the retinal pigment epithelium (RPE). The thickness of the dorsal, temporal, ventral, and nasal quadrants was calculated by averaging the thickness within a circle area 0.4 mm away from the ONH center, with a diameter of 0.4 mm. The statistical analysis was conducted using MATLAB software.

### Electroretinography Analysis

ERG measurements were carried out, as described previously,^15^ in anesthetized (100 mg/kg ketamine and 5 mg/kg xylazine) db/db and control (db/+) mice at 16 weeks and 32 weeks of age. Pupils were fully dilated with tropicamide (1%) drops and stimuli were generated and delivered using a Celeris rodent electrophysiology system (Diagnosys LLC, Lowell, MA, USA). The a-wave amplitude was measured at a fixed time following the flash to minimize post-receptor contributions to the response.^16^ The fixed time point was the same for every mouse and was selected to precede the trough, ranging from 5 to 20 ms depending on flash strength. The a-wave implicit time was measured at the trough of the response. The b-wave was measured from the trough of the a-wave to the peak of the b-wave. The b-wave implicit time was measured at the peak of the b-wave.^16^

### Fatty Acid Analysis

Extraction of the total lipids from the tissues and the preparation of methyl esters were performed as described previously.^17^ Gas chromatography/mass spectroscopy (GC/MS) analysis of fatty acid methyl esters was performed using Shimadzu QP2010 SE, equipped with a Supelco Omegawax column^18^ and 17:0 as the internal standard. Individual fatty acid values are expressed as the percentage of the total fatty acids, and the total fatty acid content was calculated using the internal standard concentration. Statistical significance was determined using Graphpad Prism 10 software. All *P* values were corrected for multiple comparisons using the false discovery rate approach.

### RNA Isolation and Quantitative Real-Time PCR 

RNA was extracted from approximately 0.3 mg mouse retina using TRIzol reagent (Thermo Fisher Scientific) and chloroform for phase separation. Due to the small amount of tissue available for RNA isolation, MB grade glycogen (Themo Fisher Scientific) was used to enhance precipitation and recovery of RNA. Following precipitation, RNA was purified using RNeasy Mini Kit (Qiagen) and treated with RNase-Free DNase (Qiagen). Purified RNA (1 µg) was then reverse transcribed using RevertAid First Strand cDNA Synthesis Kit (Thermo Fisher Scientific) and quantified via quantitative PCR (qPCR) using iTaq Universal SYBR Green Supermix (Bio-Rad Laboratories). Relative expression levels were calculated for different targets using ΔΔCT method and normalized to 18S rRNA levels. Each reaction was performed in triplicates of 10 µl final volume, where final primer concentrations were 0.625 µM for each reaction, and data were analyzed using the CFX Connect Real-Time PCR Detection System (Bio-Rad Laboratories). Primers were synthesized using the NCBI primer blast software.

Human donor peripheral retina was dissected from the retinal pigment epithelium, and total RNA was extracted and purified according to the instructions in RNeasy mini kit (Qiagen). A total of 1 µg of RNA was reverse-transcribed with a cDNA-synthesis kit (HighCapacity cDNA Reverse Transcription Kit; Thermo Fisher). The quantitative real-time PCR (qRT-PCR) assays were performed using the QuantStudioTM 7 Flex system with Fast SYBR Green Master Mix (Applied Biosystems). For normalization and relative quantification, we used CT values of the housekeeping gene β actin (ACTB). The primer sequences used for mouse and human samples are shown in Supplementary Tables S1 and S2.

## Results

### Fatty Acid Composition of Human Peripheral Retina and Macula

To determine the effects of diabetes on retinal DHA content, we analyzed the fatty acid composition of the peripheral retina and macula samples from seven diabetic donors (4 male donors and 3 female donors) and eight age-matched non-diabetic controls (5 male controls and 3 female controls). The clinical characteristics of the diabetic and control subjects are shown in the Table. No data were available for the presence of retinopathy or other eye diseases in the donors.

**Figure 1.**
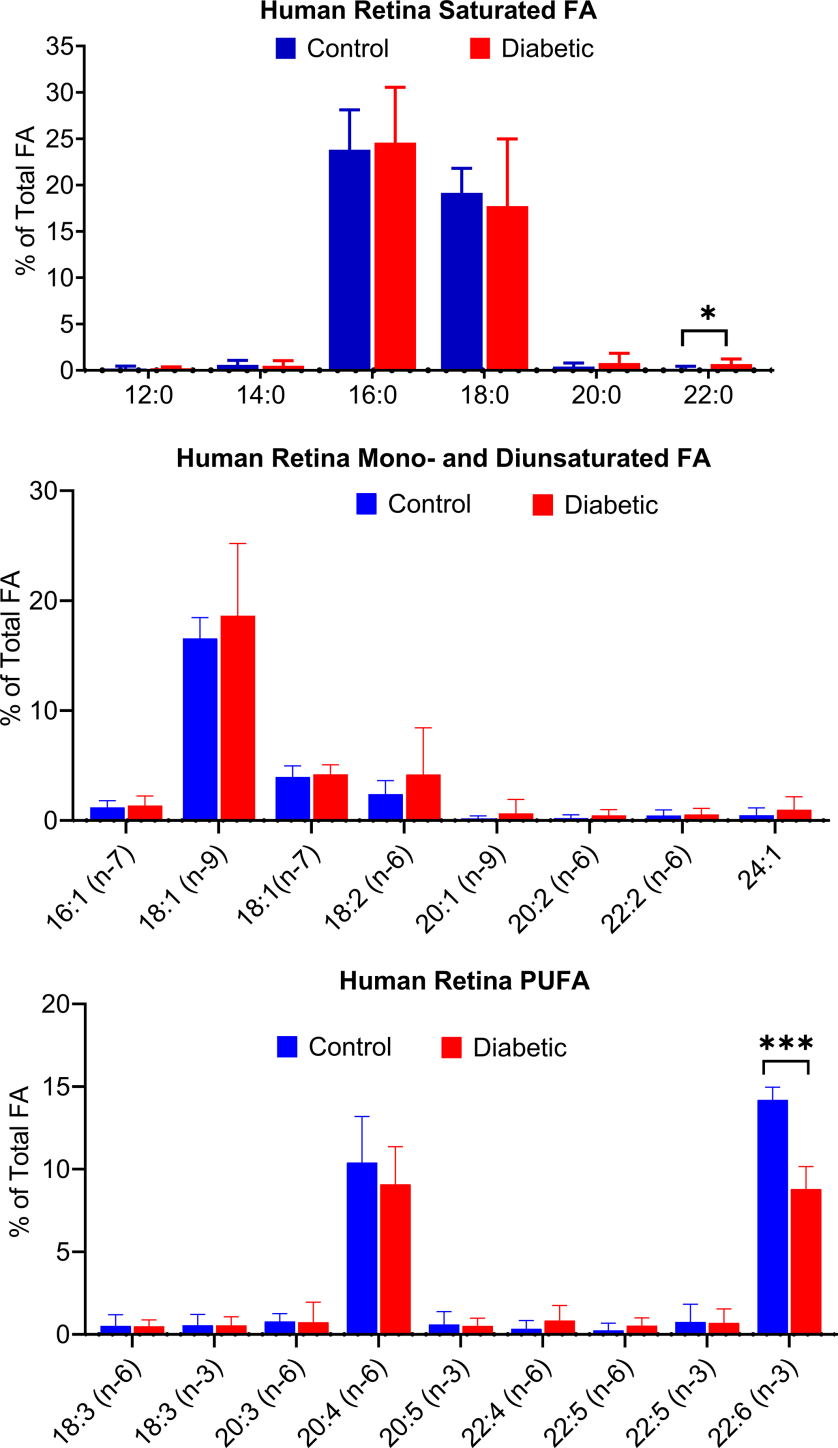
**Fatty acid composition in human peripheral retina.** Peripheral retina samples from diabetic donors (*n* = 7) and age-matched non-diabetic donors (*n* = 8) were obtained from Eversight eye bank. The fatty acid analysis was performed by GC/MS as described in the text. Statistical analysis was performed by unpaired *t* test using Graphpad Prism software. All *P* values were adjusted for multiple comparisons using the False Discovery Rate (FDR) approach. *P < 0.05; ***P < 0.0001.

Figure 1 shows the fatty acid composition in the peripheral retina samples, where we observed a 40% reduction in the DHA content of the diabetic retina compared with the non-diabetic controls (*P* < 0.0001). There was also some reduction in the content of ARA (–12%), although not statistically significant. The decrease in DHA and ARA was compensated by the increases in 18:1 and 18:2 fatty acids (not statistically significant), and in a minor saturated fatty acid, 22:0 (*P* < 0.05). The total fatty acid content was not significantly different between the diabetic donors and non-diabetic donors (data not shown).

**Figure 2.**
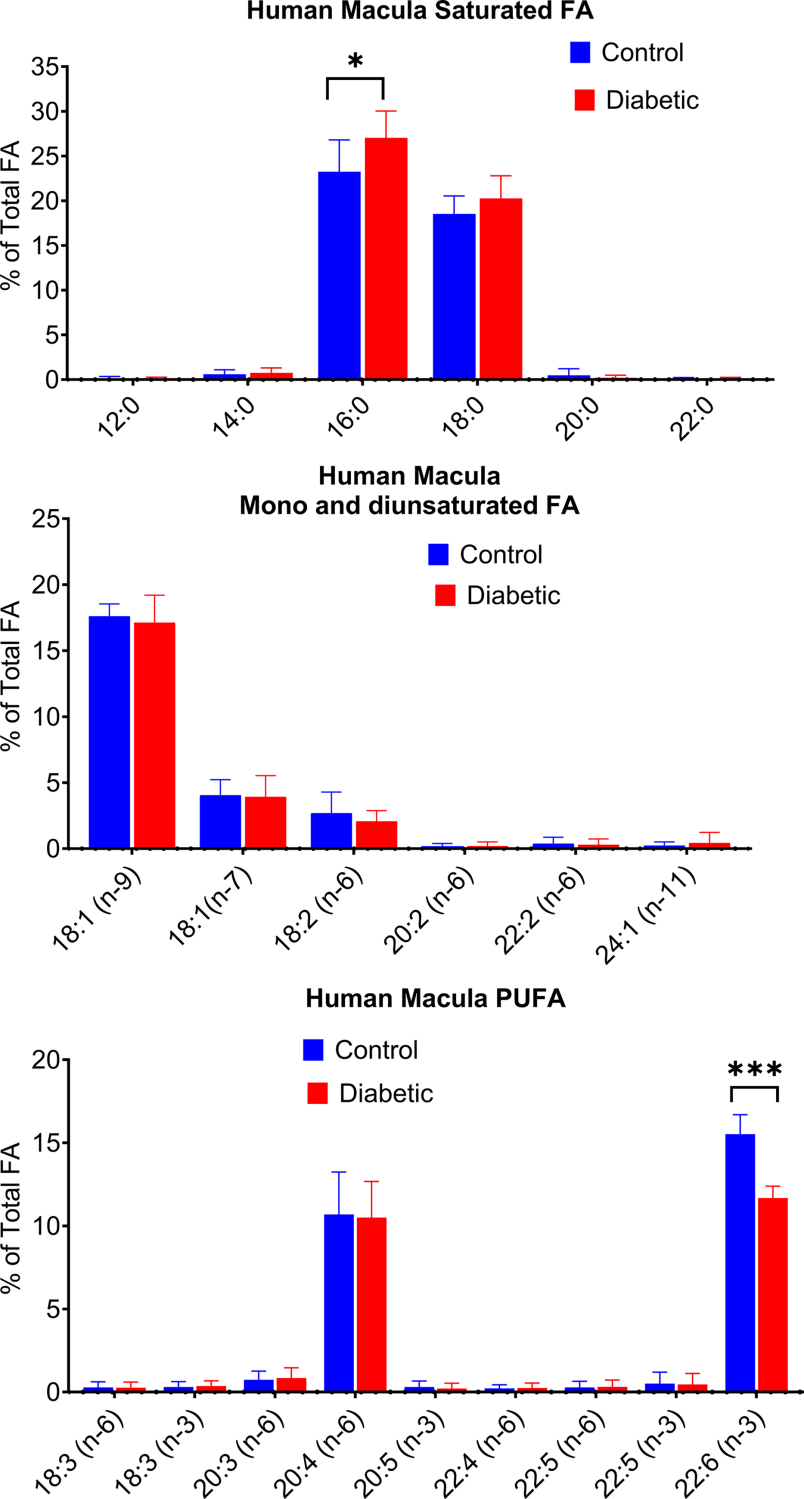
**Fatty acid composition of human macula.** The macula samples from diabetic donors (*n* = 7) and non-diabetic controls (*n* = 8) were obtained from Eversight eye bank. The analysis of fatty acids was performed by GC/MS as described in Methods. *P < 0.05; ***P < 0.0001 by unpaired *t* test (adjusted for multiple comparisons using FDR).

In Figure 2, we present the fatty acid composition of macula samples. Although a previous study^19^ reported a lower DHA content in the macula compared to the peripheral retina, we did not find such difference in our samples. However, the diabetic samples showed a significant reduction (−25%) in the DHA content (*P* < 0.0001), although the reduction was less than that found in the peripheral retina. Unlike in the peripheral retina, there was a compensatory increase in 16:0 (+16%), the major saturated fatty acid (*P* < 0.05), but no change in the ARA content. Collectively, these results show that the most significant effect of diabetes in humans is the decrease in the percentage of DHA in both the peripheral retina and the macula.

### Fatty Acid Composition of Brain and Retina in Diabetic Mice

**Figure 3.**
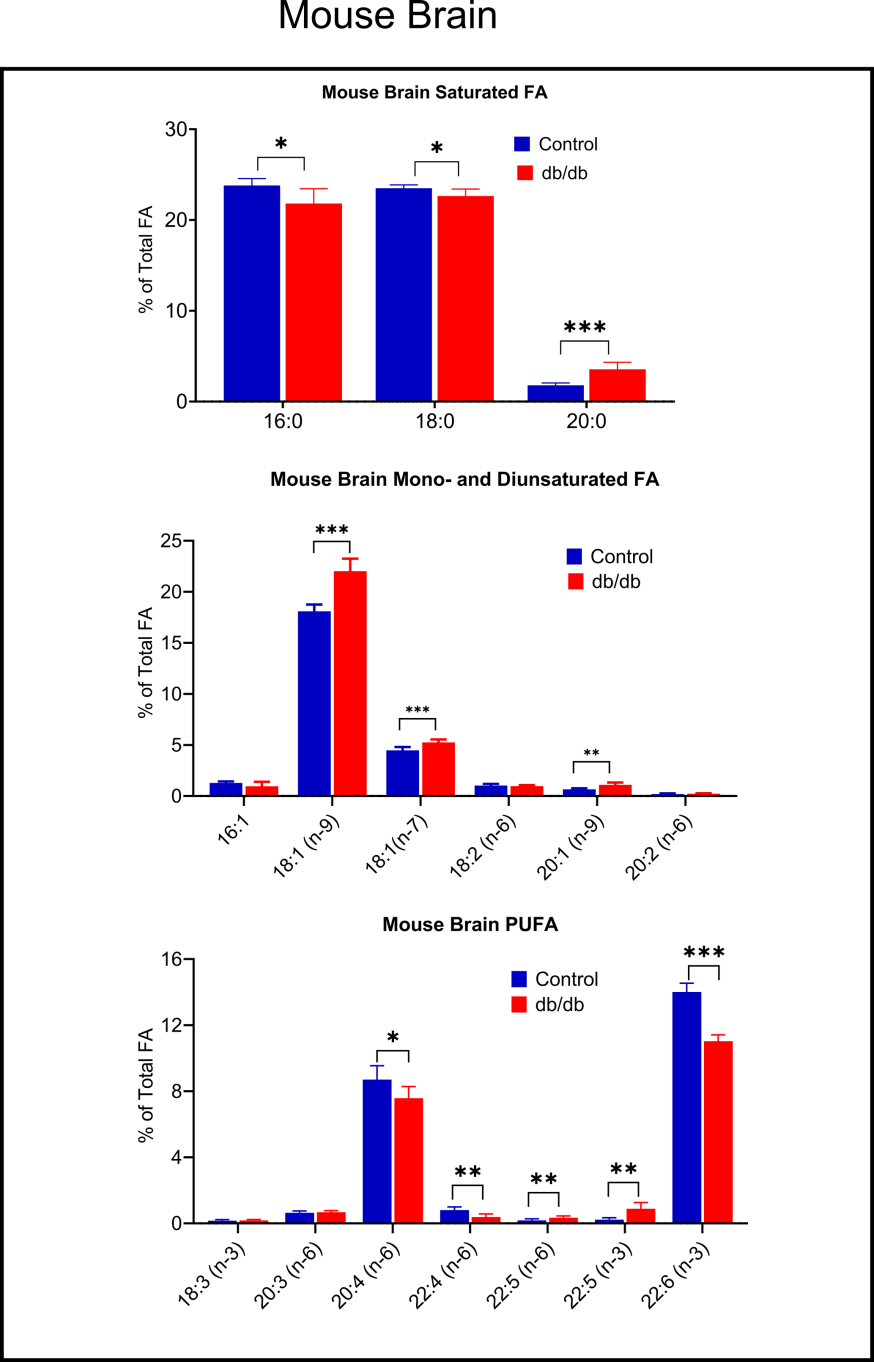
**Fatty acid composition of brain samples of db/db and age-matched db/+ mice.** Whole brain samples from 8 db/db mice and 8 db/+ littermates were analyzed for fatty acid composition by GC/MS as described in the text. *P < 0.05; **P < 0.001; ***P < 0.0001 by unpaired *t* test (adjusted for multiple comparisons using FDR).

To investigate the effect of diabetes on retinal DHA in experimental animals, we measured the fatty acid composition in the retina of db/db mice, a mouse model for type 2 diabetes. These animals spontaneously develop obesity and hyperglycemia at a young age and show other characteristics of type 2 diabetes, including diabetic retinopathy.^20^ In addition to the retina, we measured the fatty acid composition in the brain because both tissues contain high levels of DHA, and acquire DHA by a similar mechanism. Figure 3 shows the fatty acid composition in the brains of db/db mice and their non-diabetic littermates. There was a significant reduction in the levels of most of the PUFA in diabetic brains compared with the controls. DHA was reduced by 21% (*P* < 0.0001) whereas ARA was reduced by 13% (*P* < 0.05). Interestingly, there was also some reduction in the major saturated fatty acids 16:0 (−8%, *P* < 0.05) and 18:0 (−4%, *P* < 0.05). There were compensatory increases in the monounsaturated fatty acids 18:1 (n-9, +22 %, *P* < 0.0001), 18:1 (n-7, *P* < 0.001, +17%), and 20:1 (n-9, +68%, *P* < 0.05), as well as a minor saturated fatty acid 20:0 (+98%, *P* < 0.05).

**Figure 4.**
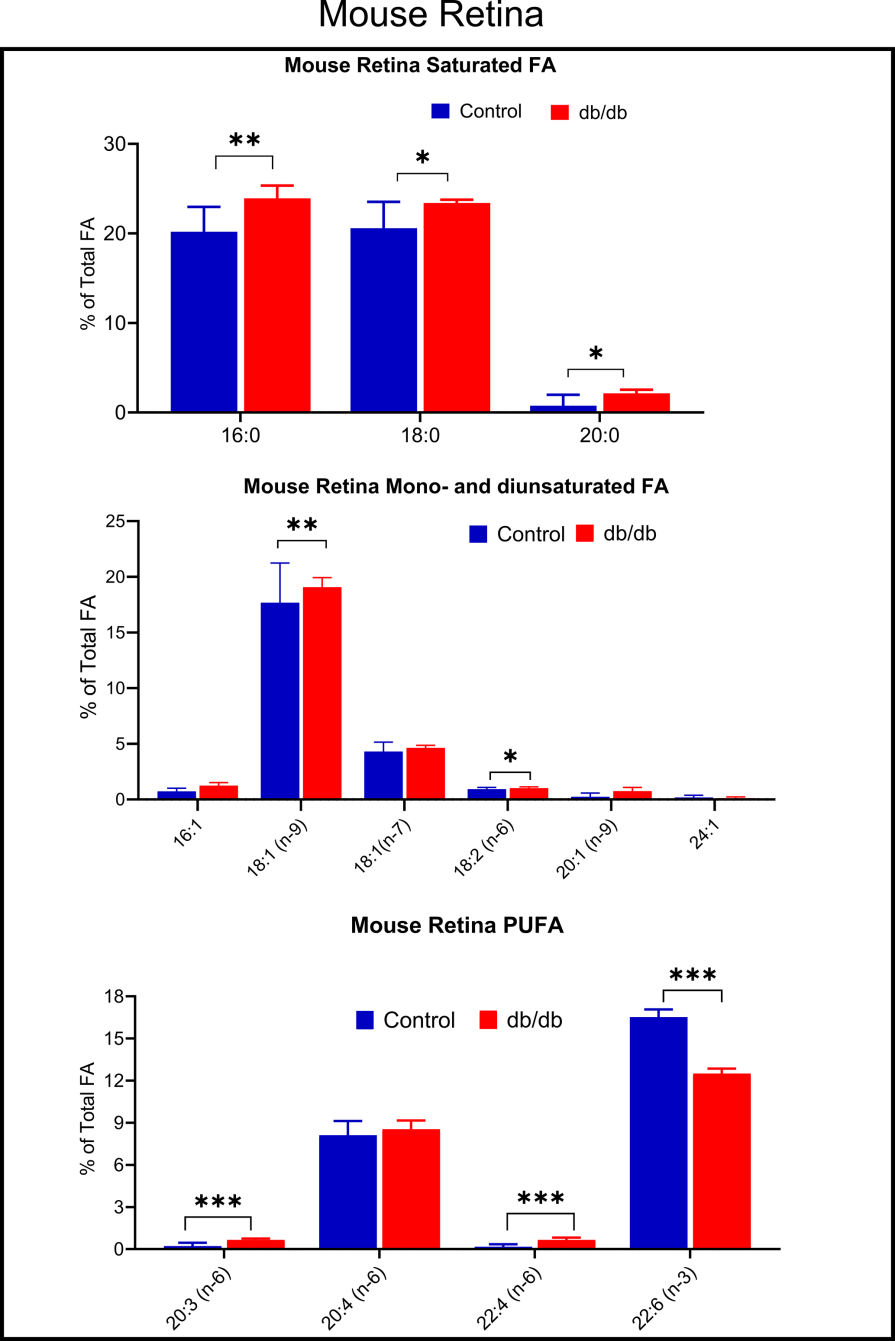
**Fatty acid composition of retina samples from db/db and age-matched db/+ mice.** Retina samples from db/db (*n* = 8) and db/+ (*n* = 8) were analyzed for fatty acid composition by GC/MS as described in the text. *P < 0.05; **P < 0.005; ***P < 0.0001 by unpaired *t* test (adjusted for multiple comparisons using FDR).

The retinas from diabetic mice showed a significant reduction in DHA compared to the retinas from the age-matched controls (−24%, *P* < 0.0001; Fig. 4). However, unlike in the brain, there was no reduction in ARA, and instead there were significant increases in the minor omega 6 fatty acids. Thus 20:3 (n-6) increased by 3-fold and 22:4 (n-6) increased by 4-fold. There were also significant increases in the 2 major saturated fatty acids 16:0 (+18%, *P* < 0.005), 18:0 (+14%), 20:0 (+180%, *P* < 0.05), and in 18:1 (n-9, +8%, *P* < 0.0001), similar to the human diabetic macula. The total fatty acid content was not different between diabetic subjects and control subjects (data not shown).

### Effect of Diabetes on Retinal Structure and Function

#### Retinal Thickness

**Figure 5.**
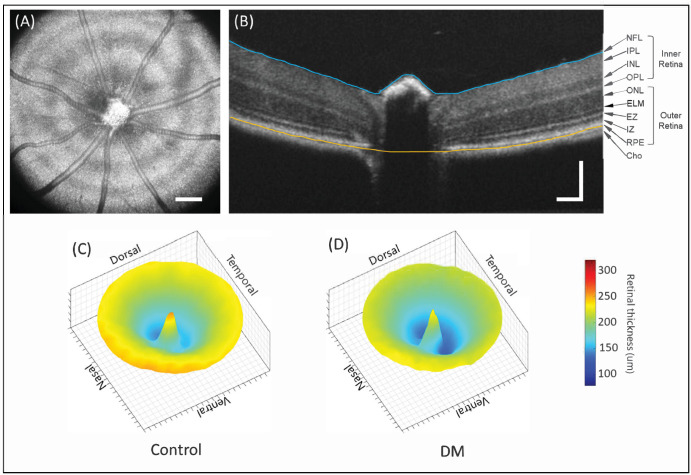
**Retinal OCT scans from db/db and db/+ mice.** (**A**) Representative enface image; (**B**) Flattened B-scan; (**C**) Average thickness map of control (db/+) mice; and (**D**) Average thickness map of db/db mice. The *scale bar* is 100 µm.

**Figure 6.**
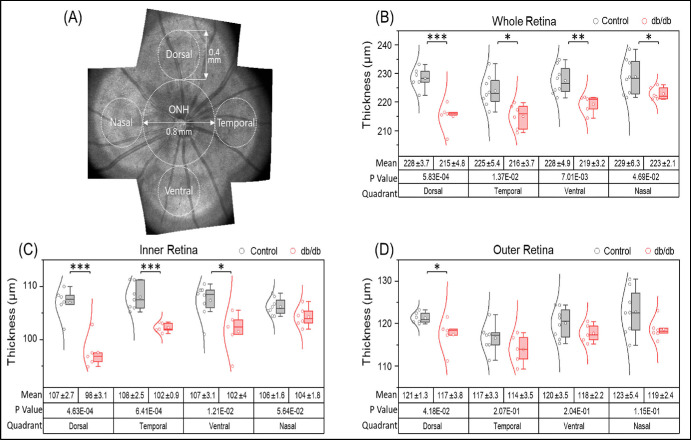
**Retinal thickness values of db/+ (control) and db/db mice as determined by OCT.** (**A**) Retina enface image montage showing the measured retinal regions. (**B**) Statistical analysis of thickness of whole retina in four quadrants. (**C**) Statistical analysis of inner retina in four quadrants. (**D**) Statistical analysis of outer retina in four quadrants. The boundaries of boxes indicate the 25th and 75th percentiles, and the horizontal line is the median value. The circle marker within the box is the mean value. Data points are distributed on the left side of the box, with a Gaussian fitting curve overlaid. The *P* values were determined by MATLAB software. *P < 0.05, **P < 0.01, and ***P < 0.001.

The OCT was used to measure the retinal thickness in db/db and db/+ mice at 32 weeks of age. Figure 5 shows the representative OCT en face image of the retina and the segmented image, as well as the representative thickness maps for db/db and control mice. The thickness map (Fig. 6) shows a significant reduction in the retinal thickness of db/db mice compared to the age-matched non-diabetic controls, around the ONH region. We have also measured the thickness in different quadrants, and the statistical analysis confirmed the retinal thickness is reduced in most quadrants (see Fig. 6B). The reduction in thickness is not symmetric, however, because the dorsal quadrant showed more thinning whereas the nasal quadrant showed less thinning (see Figs. 6C, 6D). The results also indicated that the inner retina had more thinning than the outer retina. These results agree with studies of Sheskey et al.^21^ who reported a greater decrease in the inner retina than in the outer retina of db/db mice, although Azad-Leibovich et al.^22^ reported that diabetic mice had greater retinal thickness than non-diabetic mice.

#### Electroretinography

**Figure 7.**
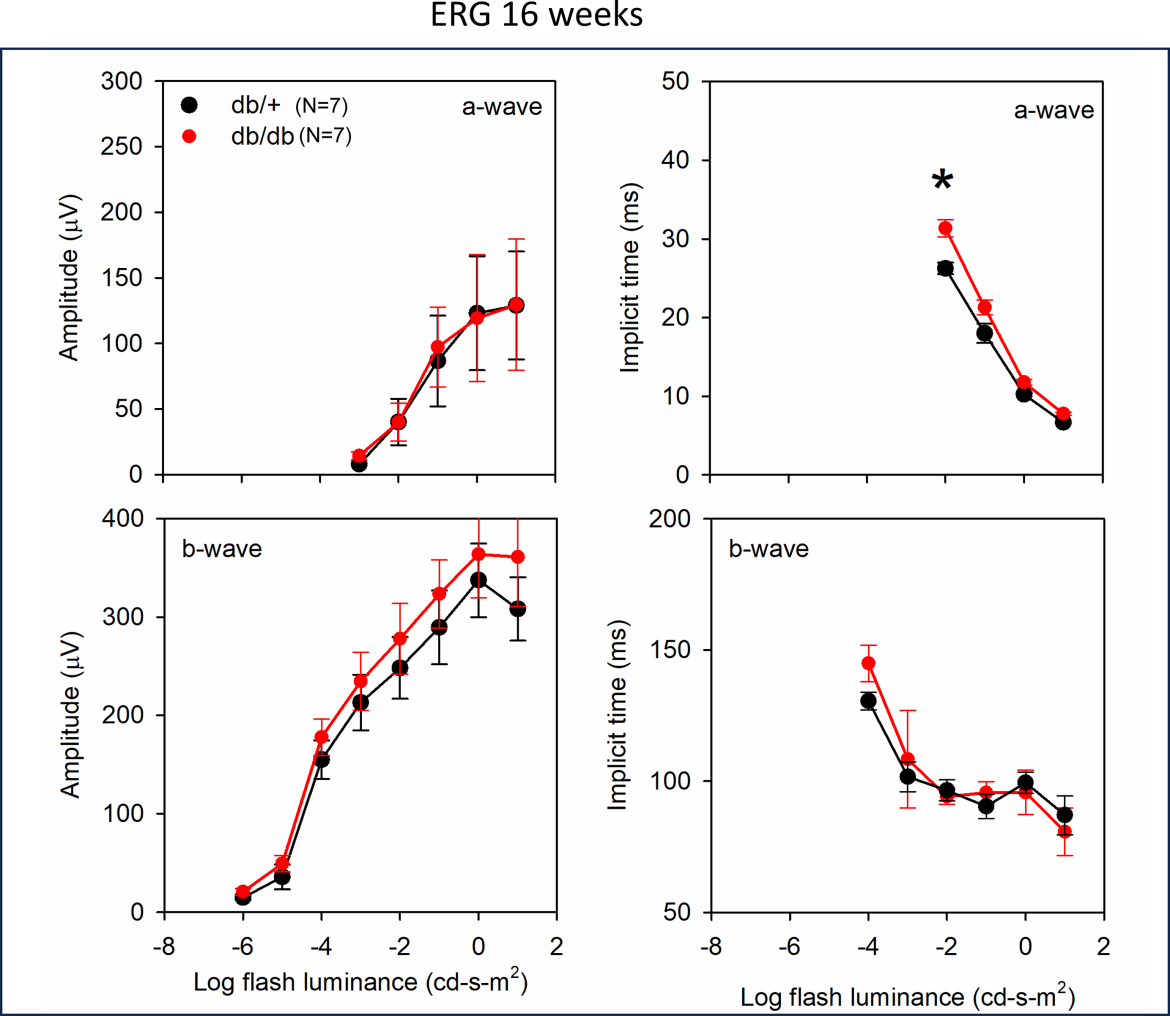
**Electroretinogram (ERG) measurements in db/+ and db/db mice at 16 weeks of age (*n* = 7 for each).** Mean amplitudes for a-wave and b-wave and their implicit times are shown. The mean ERG wave forms for db/+ and db/db mice at various luminance values are shown in Supplementary Figure S1.

Because previous studies^20^^,^^23^ have shown beneficial effects of dietary DHA on retinal function, we determined the effect of DHA deficiency on retinal function in diabetic mice, by performing ERG measurements at 16 weeks and 32 weeks of age. Mean ERG waveforms for db+ and db/db mice are shown in Supplementary Figures S1 and S2. As shown in Figure 7, there was no significant difference between diabetic and age-matched control mice at 16 weeks in the a-wave amplitude. For the a-wave implicit time, ANOVA indicated a significant effect of the group (F = 8.92, *P* = 0.01) and the interaction between the group and the flash luminance was significant (F = 7.35, *P* < 0.001). Holm-Sidak pairwise comparisons indicated significant a-wave timing differences between the db/db and the db/+ (control) groups only for the 0.01 flash luminance level (*t* = 4.74, *P* < 0.01). For the b-wave amplitude, ANOVA indicated no significant difference between groups (F = 0.48, *P* = 0.50) and no significant interaction between the group and the flash luminance level (F = 0.27, *P* = 0.96). For the b-wave implicit time, ANOVA indicated no significant differences between the groups (F = 0.02, *P* = 0.90) and the interaction between the group and the flash luminance was not significant (F = 1.32, *P* = 0.26). Thus, at 16 weeks, the ERGs for the db/db and db/+ groups were similar, except for a small timing delay of the a-wave for the db/db group.

**Figure 8.**
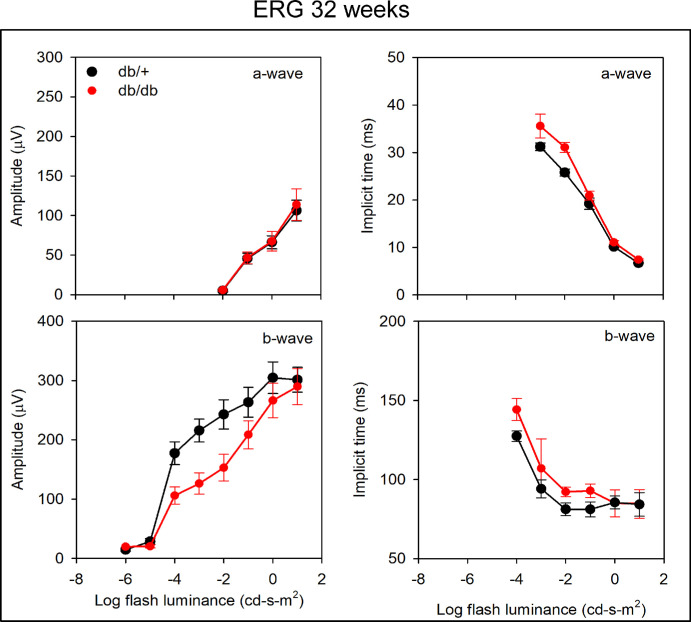
**ERG measurements in db/+ and db/db mice at 32 weeks (*n* = 7 for each).** Mean amplitudes for a-wave and b-wave and their implicit times are shown. The mean ERG wave forms for db/+ and db/db mice are shown in Supplementary Figure S2. No significant differences were observed for the a-wave amplitudes, but significant differences were observed for b-wave amplitudes at 3 different luminance values. Significant a-wave timing difference was found at the two highest flash luminance levels.

For the mice at 32 weeks (Fig. 8), ANOVA indicated no significant effect of the group for the a-wave amplitude (F = 0.20, *P* = 0.67) and the interaction between the group and the flash luminance was not significant (F = 0.40, *P* = 0.75). For the a-wave implicit time, ANOVA indicated a significant effect of the group (F = 8.92, *P* = 0.01) and the interaction between the group and the flash luminance was significant (F = 7.35, *P* < 0.001). Holm-Sidak pairwise comparisons indicated significant a-wave timing differences between the db/db and db/+ groups for the flash luminance levels of 0.01 and 0.1 cd-s-m^2^ (both *t* > 2.89, *P* < 0.01). For the b-wave amplitude, ANOVA indicated no significant difference between the groups (F = 3.44, *P* = 0.09), but there was a significant interaction between the group and the flash luminance level (F = 4.05, *P* < 0.001). Holm-Sidak pairwise comparisons indicated significant differences between the diabetic and age-matched control groups for the flash luminance levels of 0.0001, 0.001, and 0.01 cd-s-m^2^ (all *t* > 2.40, *P* < 0.03). For the b-wave implicit time, ANOVA indicated no significant differences between the groups (F = 2.68, *P* = 0.13) and the interaction between the group and the flash luminance was not significant (F = 0.72, *P* = 0.66). Thus, at 32 weeks, significant ERG abnormalities were apparent for the db/db group. The b-wave was attenuated, indicating post-photoreceptor dysfunction in these mice.

### Effect of Diabetes on Retinal Gene Expression

**Figure 9.**
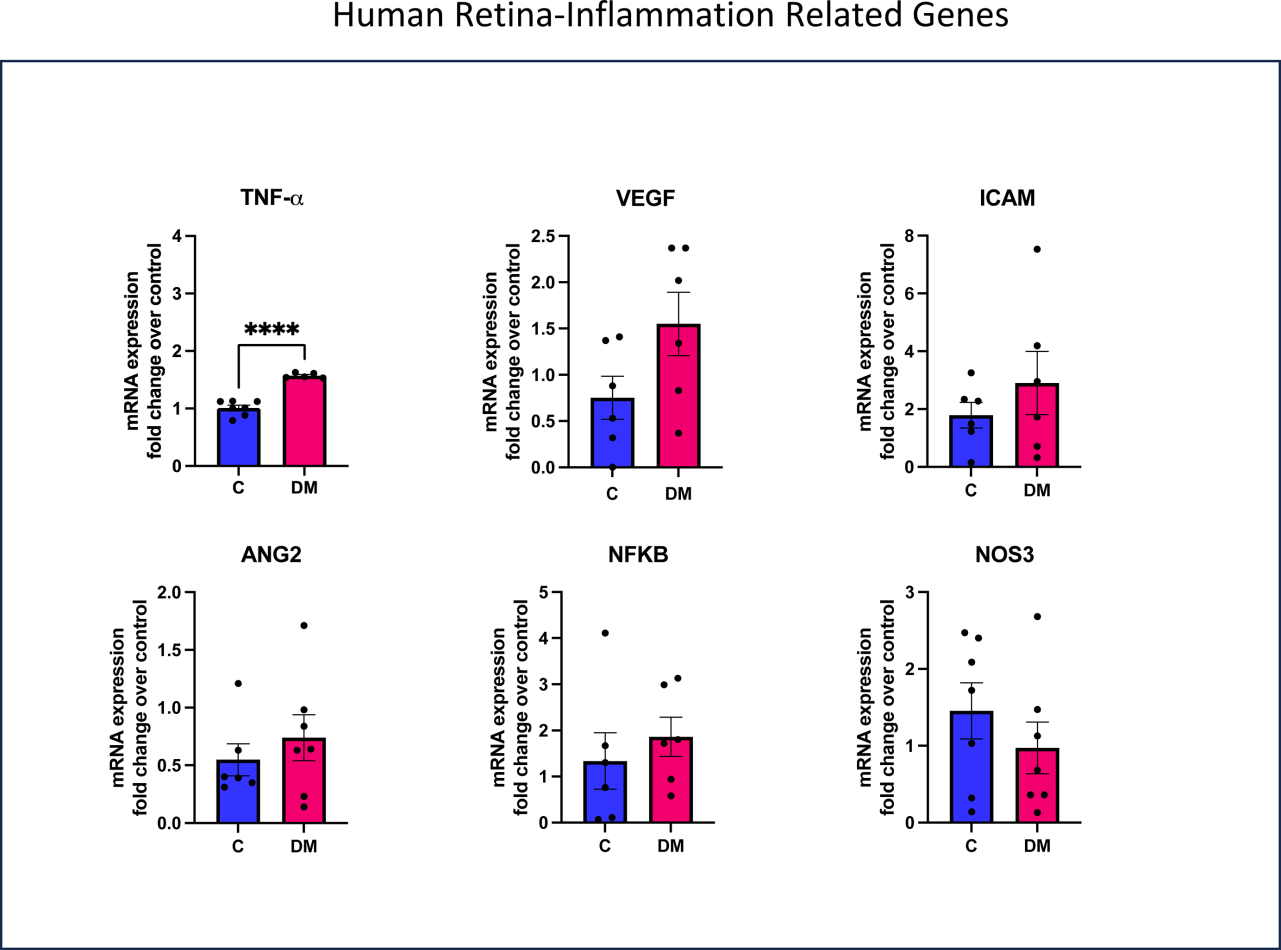
**qRT-PCR analysis of selected inflammation-related genes in human retina.** The results shown are expression levels relative to that of β-actin control. ****P < 0.0001 by unpaired *t* test.

**Figure 10.**
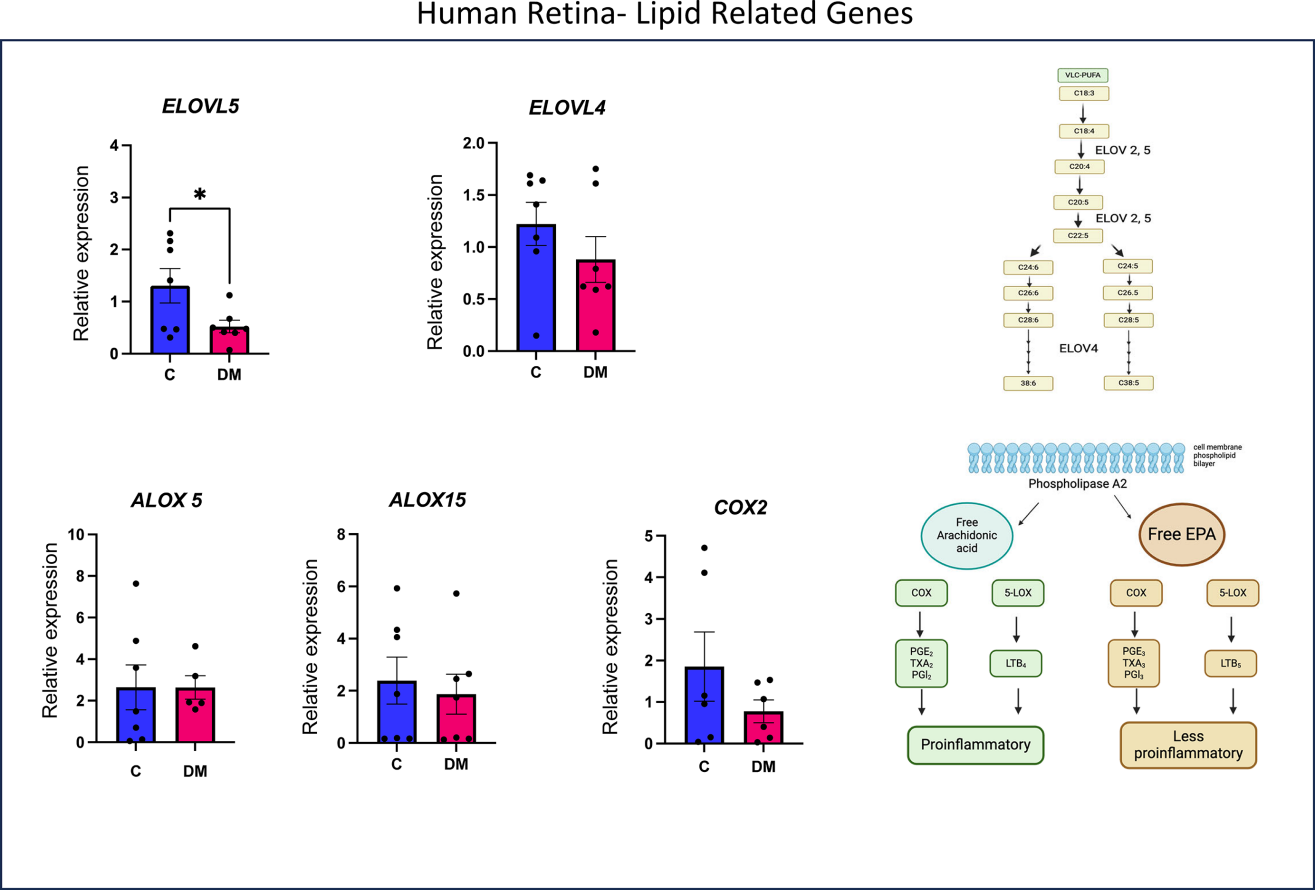
**qRT-PCR analysis of selected lipid metabolism-related genes in human retina.** The results shown are expression levels relative to that of β-actin control. The inset shows the location of the candidate genes in lipid metabolic pathways. *P < 0.05 by unpaired *t* test.

Expression of selected genes was determined by RT-PCR in human and mouse retina samples to determine whether the genes involved in inflammation and fatty acid metabolism are affected by diabetes. As shown in Figure 9, the retina from humans with diabetes showed a significant increase in the expression of the pro-inflammatory TNF-α. There were also increases in VEGF, ICAM, ANG2, and NFkB but the differences between the controls and patients with diabetes did not reach statistical significance. Figure 10 shows expression of selected genes involved in fatty acid metabolism in the human retina. Diabetic retinas showed a significant reduction in ELOV5, the enzyme responsible for the synthesis of DHA from precursors. ELOV4, involved in the synthesis of very long PUFA, and COX2, which is involved in prostaglandin synthesis, also showed some decreased expression, although not statistically significant. No significant changes were observed in the expression of the lipoxygenase genes ALOX5 and ALOX15.

**Figure 11.**
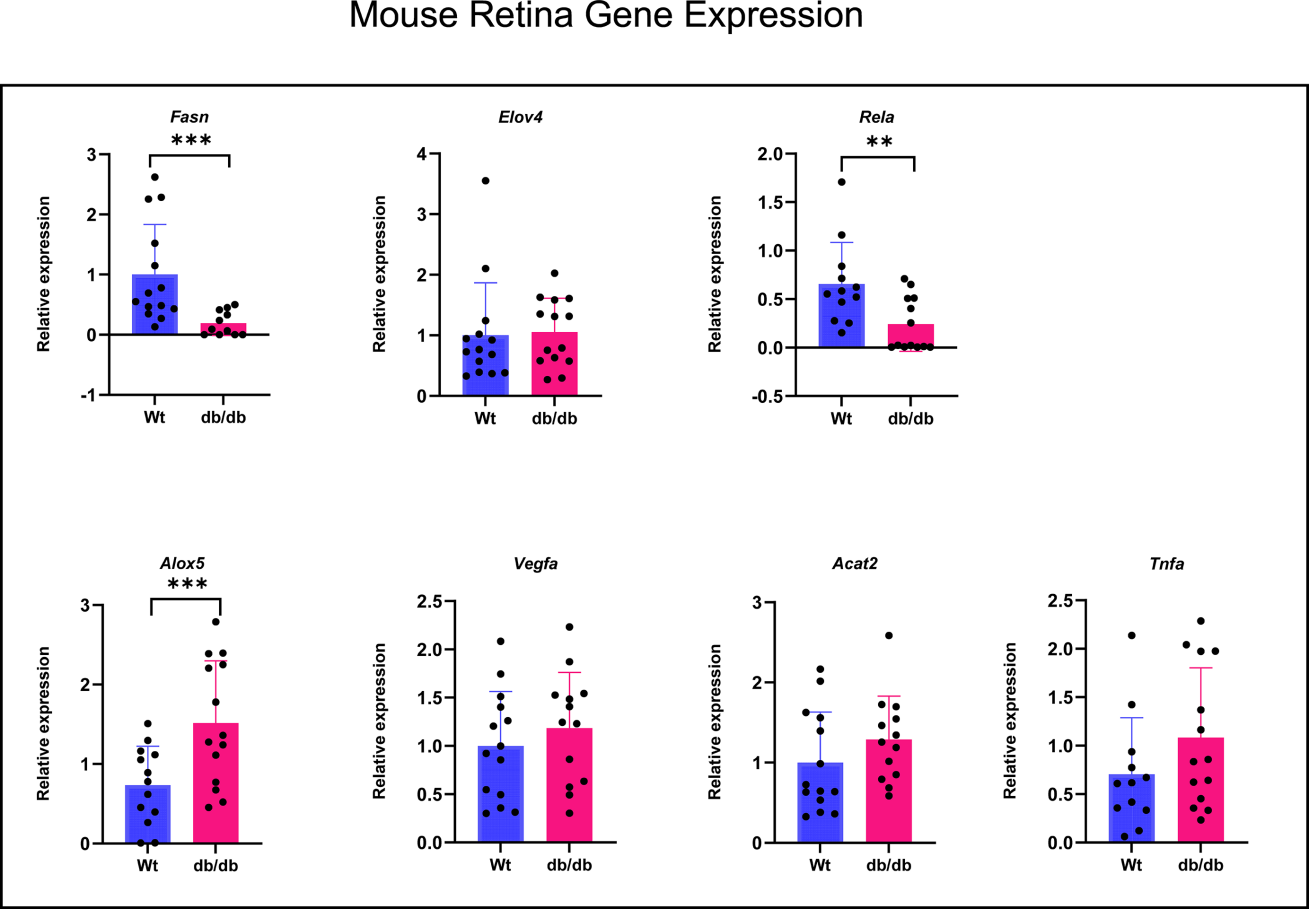
**qRT-PCR analysis of selected genes in mouse retina.** The results shown are expression levels relative to the control (18S). Statistical analysis was performed by unpaired *t* test with GraphPad software. **P < 0.01; ***P < 0.005 by unpaired *t* test.

Figure 11 shows expression of selected genes in diabetic and control mouse retinas. Here, we found a significant decrease in the expression of fatty acid synthase (FASN), the enzyme responsible for de novo synthesis of fatty acids, and a significant increase in the expression of ALOX5, which is involved in the production of pro-inflammatory leukotrienes from ARA. However, the expression of RELA, the p65 subunit of NFkB, is reduced in db/db mice compared with the controls. Although the TNFα expression is increased, the differences between control and db/db mice did not reach statistical significance.

## Discussion

Diabetic retinopathy is a major complication of diabetes and is the most common cause of blindness in the working age population. The International Diabetes Foundation estimates that global population of individuals with diabetes will reach 700 million by year 2045, about one-third of whom will develop some level of retinopathy.^24^ Although several epidemiologic studies indicated that dietary intake of omega 3 fatty acids has a negative correlation with the incidence of retinopathy, there are no data showing that diabetes causes a decline in retinal DHA. However, a reduction in the circulating levels of DHA has been reported in subjects with diabetes.^7^ The results presented here show, we believe for the first time, that the DHA content is significantly reduced in the peripheral retina and macula of subjects with diabetes, suggesting that a reduction in retinal DHA could play a role in the development of diabetic retinopathy. This possibility is supported by our data in mice, which show that the DHA content of the retina is significantly reduced in type 2 diabetic mice, which is accompanied by functional defects in diabetic mice as determined by ERG, as well as a reduction in retinal thickness determined by OCT.

There are several possible reasons for the depletion of DHA in the diabetic retina. First, hyperglycemia is known to increase the production of superoxide by enzymatic as well as non-enzymatic pathways,^14^ which could cause the oxidation of highly unsaturated fatty acids, such as DHA. The retina is particularly vulnerable to oxidative stress because of the high concentration of DHA, its elevated rate of oxygen consumption, and the exposure to direct light. Diabetes is also associated with inflammation which increases oxidative stress and the destruction of PUFA. Second, the synthesis of DHA in the retina may be impaired in diabetes, because the expression of ELOVL2, which is needed for the conversion of EPA to DHA, has been shown to be reduced by >25% in diabetic mice compared to the non-diabetic controls.^13^ Third, the uptake of DHA from circulation may be reduced in diabetes because the expression of Mfsd2a, the transporter responsible for the accretion of DHA, is significantly reduced in the diabetic mouse retina.^25^ Interestingly, we found a reduction in brain DHA also in the db/db mice supporting this possibility because both the retina and the brain are highly dependent on the Mfsd2a pathway for the accretion of DHA. The expression of Mfsd2a has also been shown to be decreased in the placenta in gestational diabetes.^26^ Moreover, overexpression of Mfsd2a, combined with increased dietary DHA reduced neovascularization in the diabetic mice.^25^

Although we cannot conclude from our data that the loss of DHA causes diabetic retinopathy in humans or mice, there is strong evidence that replenishing the retinal DHA prevents the functional defects in diabetic mice. Specifically, Sapieha et al.^20^ reported that omega 3 fatty acid supplementation improved glucose tolerance and preserved the retinal function in db/db mice. Tikhonenko et al.^23^ showed that omega 3 fatty acids prevented diabetic retinopathy in another model of type 2 diabetes (Zucker diabetic rats). Studies by Prokopiou et al.^27^ showed that the age-associated retinal degeneration, including the thinning of the retina, is prevented by omega 3 fatty acid supplementation in non-diabetic mice. Furthermore, dietary supplementation with DHA has been shown to prevent the accumulation of the toxic lipofuscin in ELOVL4 deficient mice.^28^ Supplementation with a high dose of DHA has also been reported to significantly improve macular sensitivity in diabetic patients with nonproliferative retinopathy.^11^

It is known that the retinal thickness is reduced in both patients with type I and type II diabetes,^29^ as well as in experimental diabetes.^21^ The inner retina is apparently affected more than the outer retina, as confirmed by our data in the mice. Interestingly, Arnal et al.^30^ reported that dietary DHA and lutein prevented the thinning of the retina in diabetic rats. The mechanism by which retinal thickness is increased by DHA is not yet known. However, in vitro studies show that DHA prevents apoptotic cell death of photoreceptor cells induced by oxidative stress.^31^

Our results show that the b-wave ERG amplitude is significantly decreased in 8-month-old db/db mice, whereas the a-wave amplitude was not affected, indicating a post-photoreceptor dysfunction and a possible neurodegenerative effect. This defect was not apparent at 4 months, indicating an age-dependent effect of diabetes on retinal function. Becker et al.^32^ also reported no reduction in a-wave or b-wave amplitude at 3 months, but a significant reduction of both amplitudes at 6 months of age. Samuels et al.^33^ reported b-wave amplitude losses in db/db mice as early as 2 months, followed by progressive amplitude attenuation. In addition to the b-wave amplitude loss, the ERG waveforms shown in Supplementary Figures S1 and S2 indicate a possible delay in the onset of the b-wave, which is most apparent for moderate flash strengths (–4 to –2 log cd-s-m^2^). A delay in b-wave onset would affect the shape of the a-wave trough, possibly accounting for the a-wave delays shown for moderate flash strengths in Figures 7 and 8. A delay in the onset of the b-wave is also apparent for a moderate flash intensity in previous work^34^ and a-wave delays have also been reported in db/db mice.^32^ The significantly delayed a-wave trough time for our db/db mice can likely be attributed to b-wave onset (latency) defects, rather than a delay of the photoreceptor response. Additional work is needed to support this speculation. The greater b-wave amplitude abnormality compared to a-wave abnormality is consistent with the relatively larger loss of inner-retina thickness compared to outer-retina thickness in the db/db mice (see Fig. 6). The more apparent structure and function losses of the inner-retina compared to the outer-retina are somewhat surprising in light of the reduced retinal DHA content, which is essential for normal photoreceptor structure and function. It is likely that diabetes has effects on both the inner and outer retinal structure and function and reduced photoreceptor DHA could have downstream inner retina effects.

In vitro and in vivo studies have demonstrated that EPA and DHA inhibit the expression of VEGF, which plays a critical role in development of retinopathy by altering blood retinal barrier function and by promoting neovascularization,^35^^,^^36^ supporting the potential role of DHA in the prevention of retinopathy. We have previously demonstrated that retinal DHA and EPA can be more effectively increased by feeding LPC-EPA/DHA than by feeding TAG-EPA/DHA supporting the importance of the Mfsd2a pathway for the maintenance of retinal DHA levels.^37^ In addition, we showed that feeding LPC-EPA/DHA to 5XFAD mice which develop Alzheimer's disease-related retinopathy, significantly improved their retinal function.^15^ Therefore, we suggest that enriching retinal DHA through dietary LPC-EPA/DHA, the preferred substrate for Mfsd2a, may prove beneficial in the prevention of diabetic retinopathy.

## Supplementary Material

Supplement 1
