# Supplementary material for: Retinal Docosahexaenoic Acid Is Significantly Reduced in Diabetic Humans and Mice: Possible Relationship to Diabetic Retinopathy
Source: Invest Ophthalmol Vis Sci. 2024 Dec 27;65(14):39. doi: 10.1167/iovs.65.14.39 (PMC11684127; doi:10.1167/iovs.65.14.39)

Supplement Table S1<br><br>Primers for Mouse Retina			
**Gene**	**Species**	**Forward Primer 5’-3’**	**Reverse Primer 5’-3’**
Fasn	Mouse	5-CAAGTGTCCACCAACAAGCG-3	5-GGAGCGCAGGATAGACTCAC-3
Elov4	Mouse	5-GCAATGCTTGTGTCCCCTTG-3	5-TTCTGCAGTCTTCCGGTCAC-3
Rela	Mouse	5-GCCTCTGGCGAATGGCTTTA-3	5-GGTGAGGTGGATCCTTGGTG-3
Alox5	Mouse	5-CGAGTGACAGGGTCAAGAAGT-3	5-GGAACTCGATGTAGTCCCCG-3
Vegfa	Mouse	5-TATTCAGCGGACTCACCAGC-3	5-AACCAACCTCCTCAAACCGT-3
Acat2	Mouse	5-CCAACAAGTGTGCCTCTGGA-3	5-AACATCCCATCCCGTCAAGC-3
Tnfa	Mouse	5-GCCTCTTCTCATTCCTGCTTG-3	5-CTGATGAGAGGGAGGCCATT-3
18s	Mouse	5-CTCAACACGGGAAACCTCAC-3	5-AGACAAATCGCTCCACCAAC-3

Supplement Table S2

Primers for Human Retina

Gene	Species	Forward Primer 5'-3'	Reverse Primer 5'-3'
NOS3	Human	5-GTTAGATTCCTC TT CCTCTCTC-3	5-GGCACAGTCCCTTATGGTAAA- 3
VEGFA	Human	5-CAC CCA CCC ACA TAC ATA CAT-3	5-AGTCTCTCATCTCCTCCTCTTC-3
TNF	Human	5-CCAGGGACCTCTCTCTAATCA-3	5-TCAGCTTGAGGGTTTGCTAC-3
VCAM	Human	5-GATTGGTGACTCCGTCTC ATT-3	5-CCTTCCCATTCAGTGGACTAT-3
ANGPT2	Human	5-ATCAGGACACACCACGAA G-3	5-CATCCTCACGTCGCTGAATAA-3
NFKB	Human	5-CTCCACAAGGCAGCAAATAGA-3	5-ACTGGTCAGAGACTCGGTAAA-3
ICAM	Human	5-GTAGCAGCCGCAGTCATAAT-3	5-GGGCCTGTTGTAGTCTGTATTT-3
ALOX15	Human	5-CTGCGATACACCCTGGAAATTA-3	5-CACAGCCACGTCTGTCTTATAG-3
ALOX5	Human	5-GGCACTGACGACTACATCTAC-3	5-GCCGTGTTTCCAGTTCTTTAC-3
ELOVL4	Human	5-AGCTGCATGTGTAGGATGTATG-3	5-TGAAATCAGGTAGCAGGGAATG-3
ELOVL5	Human	5-CAGGAGTATGGGAAGGCAAATA-3	5-GTACATGAGGACGTGGATGAAG-3
COX2	Human	5-TGGACAACCTGTCACCTTTAC-3	5-TCACTCACCAAGGCCATTATC-3
ACTB	Human	5-GGA TCAGCAAGCAGGAGTATG-3	5-AGAAAGGGTGTAACGCAACTAA-3

Supplement Fig. S1

Waveforms for db/+ and db/db mice 16 weeks

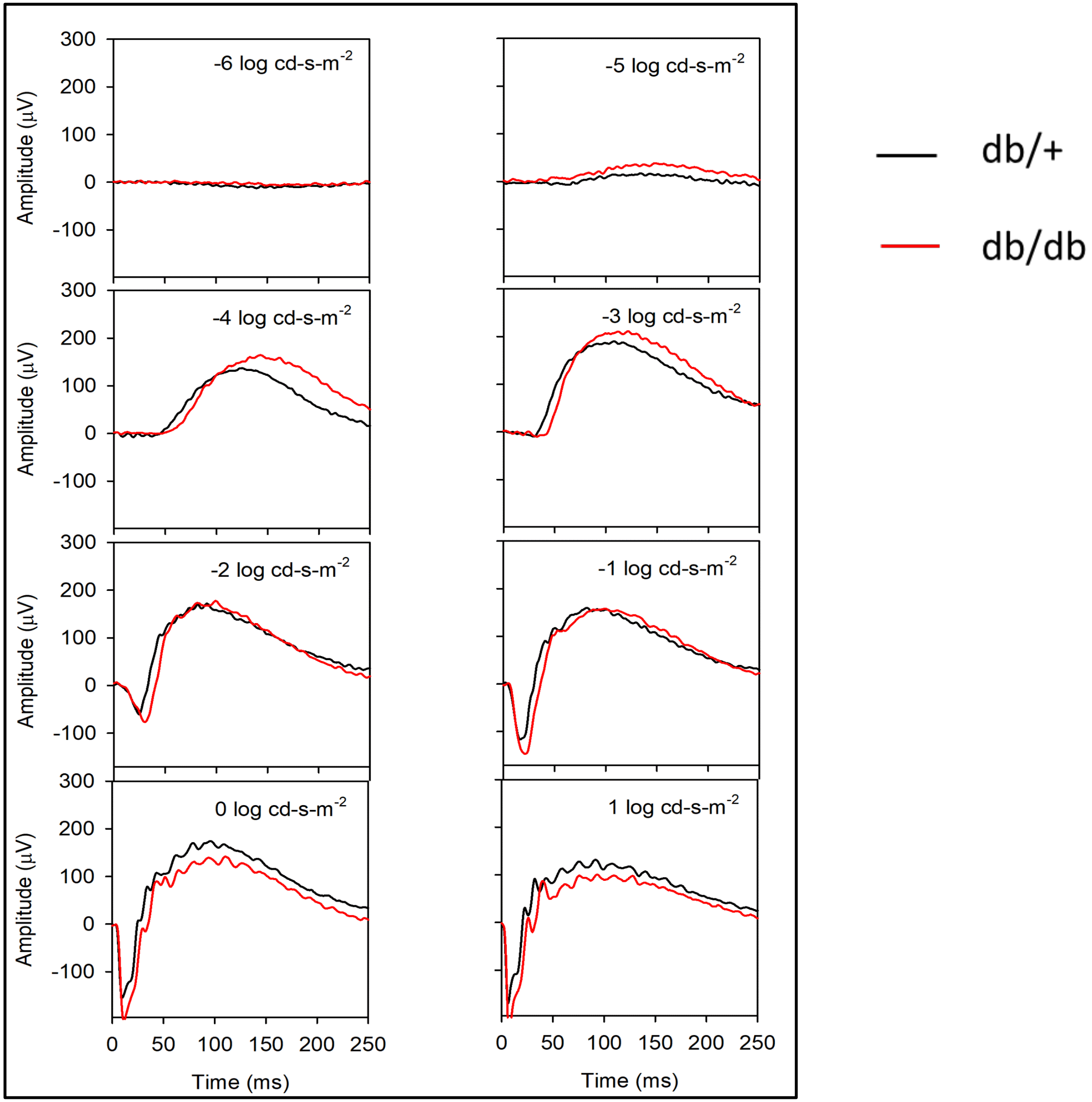

Supplement Fig.S2

Waveforms for db/+ and db/db mice 32 weeks

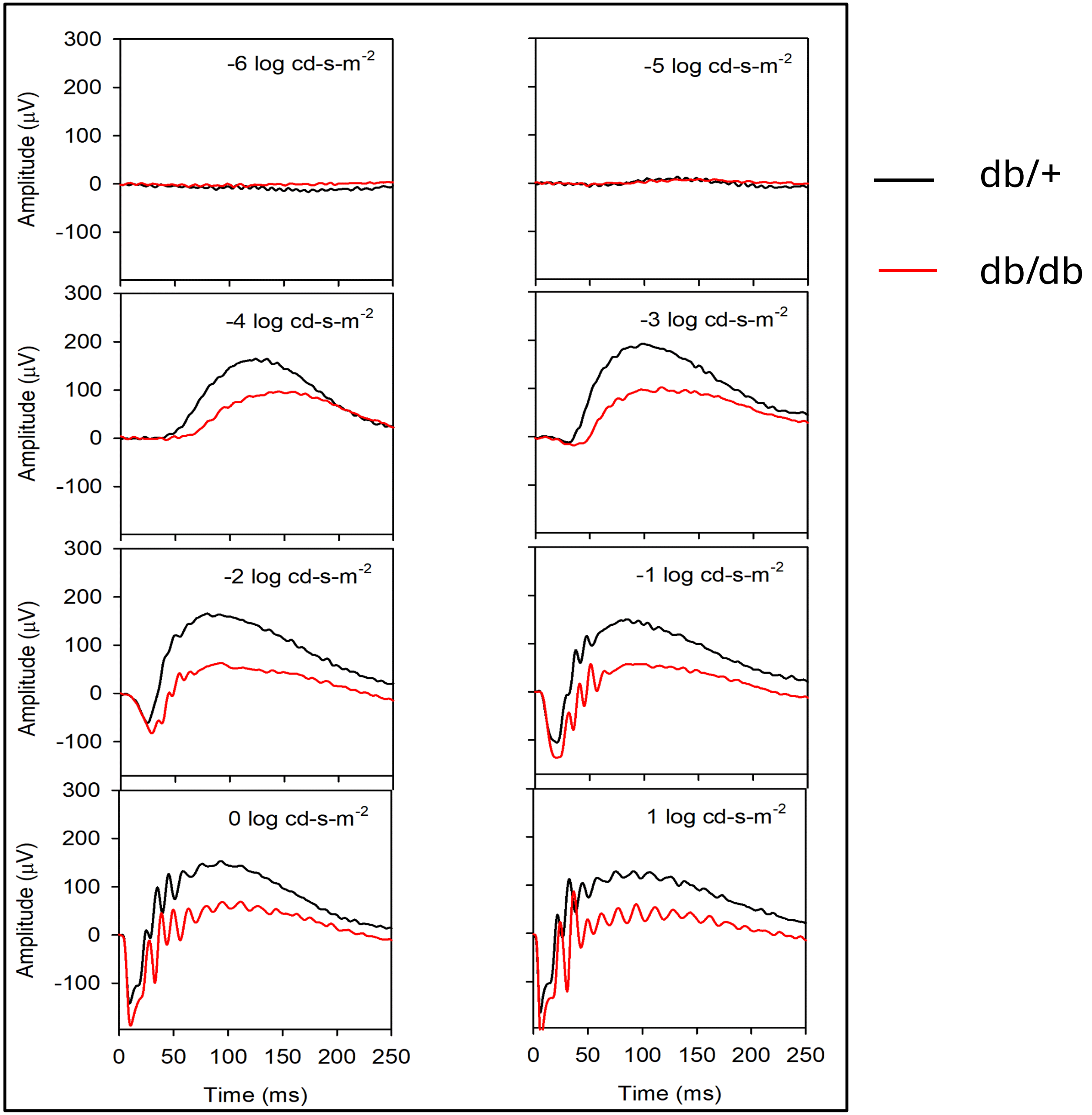

Supplement: Supplement 1 [file iovs-65-14-39_s001.pdf]
